# Hedgehog inhibitors exert anti-proliferation effects and synergistically interact with trastuzumab in HER2-positive gastric cancer models

**DOI:** 10.2340/1651-226X.2025.42219

**Published:** 2025-05-27

**Authors:** Zixin Yang, Ren Niu, Jinzhu Han, Jie Guo, Yingqian Lv

**Affiliations:** The second department of Oncology, The Second Hospital of Hebei Medical University, Hebei, China

**Keywords:** Gastric cancer, HER2 receptors, Trastuzumab, Hedgehog signalling, AKT/mTOR

## Abstract

**Background:**

Gastric cancer (GC) remains a significant health concern with limited therapeutic options. While trastuzumab, a Human Epidermal Growth Factor Receptor 2 (HER2)-targeting antibody, has shown efficacy in HER2-positive GC, its therapeutic response is moderate. Hedgehog (Hh) signalling plays a critical role in the progression of GC.

**Methods:**

We evaluated the sensitivity of various GC cell lines to trastuzumab. The HER2-positive HGC-27 cell line was identified as the most sensitive. In addition, the effects of two Hedgehog inhibitors, vismodegib and cyclopamine, were assessed on cell growth by monitoring SMO expression. Both *in vitro* and *in vivo* assays were conducted to explore the combination of Hh inhibitors and trastuzumab.

**Results:**

Both vismodegib and cyclopamine exerted anti-proliferative effects, and synergistically enhanced the anti-tumour activity of trastuzumab in HER2-positive GC models. Mechanistically, Hh inhibitors inhibited the AKT/mTOR signalling pathway through Smoothened (SMO) depletion, contributing to their anti-growth effects.

**Interpretation:**

This study highlights the potential of combining Hh inhibitors with trastuzumab as a therapeutic strategy for HER2-positive GC by targeting the AKT/mTOR pathway.

## Background

Gastric cancer (GC) ranks as the fifth leading cause of cancer-related mortality globally and is the fourth most frequently diagnosed cancer [1]. Gastric cancer is a multifactorial disease, influenced by both environmental and genetic factor [2]. The most well-recognised cause of GC is *Helicobacter pylori* infection, with Epstein-Barr virus also demonstrating an association with GC onset and development. In addition, lifestyle choices such as cigarette smoking, obesity, high dietary salt consumption, inadequate fruit and vegetable intake, and elevated consumption of preserved foods have all been correlated with an elevated susceptibility to GC [3]. According to the GLOBOCAN 2040 projection model, there are expected to be approximately 1,800,000 new cases of stomach cancer globally by 2040, with an estimated 1,300,000 deaths [4]. The current mainstream treatments for stomach cancer mainly include surgical resection, chemotherapy, and radiation therapy [5, 6]. Despite the advance in perioperative management, the clinical outcome of GC patients remains unsatisfactory, with the 5-year survival rate less than 20% [7]. Hence, more effective therapies against GC progression are urgently required.

Human Epidermal Growth Factor Receptor 2 (HER2) protein is a 185-kDa receptor tyrosine kinase playing crucial roles in the pathogenesis of multiple cancers [8–10]. Over-expression of HER2 has been found to occur in up to 23% of GC cases, and is positively correlated with tumour metastasis, high histological grade, and poor prognosis [11]. The HER2-positive GC is characterised by significant molecular and clinical heterogeneity. For instance, HER2 amplification varies spatially within tumours (intra-tumoral heterogeneity) and temporally during disease progression, impacting therapeutic responses [12]. In addition, HER2-positive GC subtypes exhibit divergent signalling pathway activation (e.g. PI3K/AKT vs. MAPK), genomic instability profiles, and immune microenvironment compositions [13, 14]. Numerous efforts have been made to develop new molecular-directed cancer therapy targeting HER2. The HER2-targeting monoclonal antibody trastuzumab has shown efficacy in treating HER2-positive GC, as demonstrated by the Trastuzumab for Gastric Cancer (ToGA) trial. This phase 3, open-label, randomised controlled trial, which showed a median overall survival of 13.8 months in the trastuzumab plus chemotherapy group compared to 11.1 months in the chemotherapy-alone group, established trastuzumab in combination with chemotherapy as the first-line treatment for HER2-positive advanced GC [15, 16]. More recently, the KEYNOTE-811 trial further advanced the treatment paradigm by integrating immunotherapy with HER2-targeted therapy [17]. However, this benefit is limited to only approximately 20% of advanced GC patients, and the inevitable gaining of resistance to trastuzumab remains a great challenge in advanced GC [18]. To this end, it is urgent to develop combination regimens for HER2-positive GC patients, to increase the therapeutic efficacy of currently available treatments.

The Hedgehog (Hh) signalling pathway is an evolutionary conservative pathway that plays an indispensable role in diverse biological functions in both invertebrates and vertebrates. The canonical Hh signalling pathway involves ligand (e.g. SHH) binding to PTCH1, which relieves inhibition of SMO, leading to activation of Glioma-associated oncogene homolog (GLI) transcription factors. Non-canonical Hh signalling refers to GLI activation independent of Sonic Hedgehog/ Protein Patched Homolog/ Smoothened (SHH/PTCH/SMO), often via crosstalk with other pathways (e.g. PI3K/AKT, RAS/MAPK) [18–20]. Previous studies have confirmed the involvement of the Hh signalling pathway in preserving cell stemness, survival, cell growth, adhesion, and regulating cell death [21, 22]. The expression level of GLI was found to be upregulated in GC tissues, and this elevation is positively correlated with poor prognosis [23]. Also, nanoparticles targeting SMO were proven to notably inhibit malignant features of the human GC cell line BGC‐823 and SGC‐7901 [24]. Previous data showed that Hh signalling mediators, or in combination with classical chemotherapeutic agents, exhibit promising performance in cancer treatment, particularly in basal cell carcinoma. For example, Vismodegib (Vis) and sonidegib are Food and Drug Administration (FDA)-approved oral small-molecule SMO inhibitors used in the treatment of metastatic basal cell carcinoma. Clinical trials have shown an objective response rate of 33–67% in advanced basal cell carcinoma patients who received neoadjuvant treatment with Vis, with most patients experiencing partial or complete tumour regression [25]. In GC, as reported by Na et al., cyclopamine (Cyc), another natural small-molecule antagonist of Hh signalling, can exert anti-tumour effects by sensitising Tumour Necrosis Factor-Related (TNF)-related apoptosis-inducing ligand (TRAIL) resistant GC cells to TRAIL-mediated cell death via regulating stress response [26]. In addition, GLI overexpression in human pancreatic cancer cell lines led to a markedly invasive phenotype and Cyc showed promising outcomes when used together with conventional antimetabolites [27]. It is worth mentioning that, in the occurrence and progression of tumours, there is crosstalk between the HH signalling pathway and the HER2 gene in multiple aspects. It has been reported that Hh signalling pathway plays a critical role in maintaining stemness in trastuzumab-resistant HER2-positive breast cancer cell lines [28]. Also, both Hh and HER2 pathways converge on critical downstream effectors such as PI3K/AKT/mTOR and RAS/ERK, creating synergistic pro-tumorigenic signalling.

## Objectives

This study investigates the therapeutic efficacy of combining Hh pathway inhibitors (Vis and Cyc) with trastuzumab in HER2-positive GC cells. We hypothesise that dual targeting of Hh and HER2 signalling pathways synergistically enhances anti-tumour effects by suppressing AKT/mTOR-driven proliferation and survival. Our objectives are to:

1. Evaluate the combinatorial effects of Hh inhibitors and trastuzumab on tumour growth and apoptosis *in vitro* and *in vivo*.
2. Mechanistically define the role of AKT/mTOR signalling in mediating this synergy.

## Materials and methods

### Cell culture and transfection

Normal human gastric epithelial (GES-1) cells and three human GC cell lines (SNU-216, NCI-N87, and HGC-27) were obtained from the Type Culture Collection of the Chinese Academy of Sciences (Shanghai, China) and were grown in RPMI medium (Invitrogen, Carlsbad, CA, USA; Cat# 11875093) containing 10% foetal bovine serum (FBS) (Invitrogen, Carlsbad, CA, USA; Cat#10099141) and 1× penicillin-streptomycin (Biofluids Inc., Camarillo, CA, USA; Cat#P4333). All cells are kept at 37°C (5% CO2). To evaluate the effects of SMO overexpression on the cell growth, a total of 100 μg SMO overexpression plasmids (pCMV3-SMO, Sino Biological, catalogue no. HG11711-CF; backbone vector: pCMV3) were transfected into HGC-27 cells using Lipofectamine 3000 (Invitrogen, Carlsbad, CA, USA; Cat#L3000008) according to the manufacturer’s instructions. The plasmid encodes the full-length human *SMO* transcript (RefSeq NM_005631.4). The construct was validated by sequencing prior to transfection. 48 h post-transfection, the cells were used for EdU assay, colony formation assay and Western blot analysis.

### EdU assay

The EdU assay was conducted following the manufacturer’s instructions with the EdU assay Kit (RiboBio Co., Ltd., Guangzhou, China; Cat#C10310-3). Briefly, the assay was performed in 24-well plate (50,000 cells per well), with EdU reagent being added to proliferating cells for 2 h. HGC-27 cells were fixed with 4% paraformaldehyde for 30 min at room temperature. Subsequently, cells were stained with Apollo Dye and protected from light. Then, nucleic acids were stained with Hoechst for 30 min at room temperature. Images were captured under a microscope (Zeiss, AX10, Germany), and the percentage of EdU-positive cells was assessed by counting an average of 500–1,000 cells per field in three randomly chosen sample regions, employing ImageJ software (version 1.53t; National Institutes of Health, USA; https://imagej.nih.gov/ij/) [29].

### Colony formation assay

Colony formation assay was performed as previously described [30]. Approximately 200 GC cells were plated in six-well dishes and cultured for 11 days. The medium was replaced every 3 d. Following washing, fixation, and staining with 0.01% crystal violet solution, the cell colonies (containing more than 50 cells) were photographed and assessed with Quantity One software (version 4.6.9; Bio-Rad Laboratories, Inc., USA).

### Migration and invasion assays

#### Migration assay

The migration ability of HGC-27 cells was evaluated using a transwell migration assay. Briefly, HGC-27 cells were seeded in the upper chamber of a 24-well transwell plate (8 μm pore size, Corning, USA) at a density of 5 × 10^4 cells per well in serum-free Roswell Park Memorial Institute (RPMI) medium. The lower chamber was filled with RPMI medium containing 10% FBS as a chemoattractant. After 24 h of incubation at 37°C, the cells that migrated through the membrane were fixed with 4% paraformaldehyde for 30 min and stained with 0.1% crystal violet for 20 min. The migrated cells were then counted under a light microscope (Zeiss, AX10) in five randomly selected fields.

#### Invasion assay

The invasion ability of HGC-27 cells was assessed using a Matrigel-coated transwell invasion assay. The upper chamber of a 24-well transwell plate (8 μm pore size, Corning, USA) was pre-coated with Matrigel (BD Biosciences, USA) and allowed to solidify at 37°C for 1 h. HGC-27 cells were seeded in the upper chamber at a density of 5 × 10^4 cells per well in serum-free RPMI medium, while the lower chamber was filled with RPMI medium containing 10% FBS as a chemoattractant. After 48 h of incubation at 37°C, the cells that invaded through the Matrigel and membrane were fixed with 4% paraformaldehyde for 30 min and stained with 0.1% crystal violet for 20 min. The invaded cells were then counted under a light microscope (Zeiss, AX10) in five randomly selected fields.

### Western blotting

Cells were washed three times and lysed in RIPA buffer (Solarbio Life Sciences, Beijing, China; Cat#R0010) supplemented with protease/phosphatase inhibitor (Solarbio Life Sciences; Cat#P1260) to extract proteins. Equal amounts of protein (30 μg per lane) were separated on 10% SDS-PAGE (Sodium Dodecyl Sulfate-Polyacrylamide Gel Electrophoresis) gels and subsequently transferred onto PVDF (polyvinylidene fluoride) membranes. After blocking with bovine serum albumin for 2 h, the membranes were incubated with the specified primary antibodies overnight at 4°C. After that, the membranes were incubated with the indicated secondary antibody. The following primary antibodies were used: anti-HER2 (Abcam, Cambridge, UK; Cat#ab134182, 1:1000 dilution), anti-SMO (Abcam; Cat#ab124964, 1:1000 dilution), anti-GLI1 (Abcam; Cat#ab273018, 1:1000 dilution), Ki67 (Abcam; Cat#ab16667, 1:1000 dilution), Bax (Abcam; Cat#ab32503, 1:1000 dilution), Bcl-2 (Abcam; Cat#ab32124, 1:1000 dilution), Caspase-3 (Abcam; Cat#ab32351, 1:1000 dilution), Caspase-9 (Abcam; Cat#ab32539, 1:1000 dilution), anti-mTOR (Cell Signaling Technology, Danvers, MA, USA; Cat#2972, 1:1000 dilution), anti-phospho-mTOR (Cell Signaling Technology; Cat#2971; 1:1000 dilution; detects phosphorylation at serine 2448), anti-AKT (Cell Signaling Technology; Cat#9272, 1:1000 dilution), anti-phospho-AKT (Ser473) (Cell Signaling Technology; Cat#9271, 1:1000 dilution; detects phosphorylation at serine 473), anti-4EBP1 (Cell Signaling Technology; Cat#9644, 1:1000 dilution), anti-phospho-4EBP1 (Cell Signaling Technology; Cat#2855; 1:1000 dilution; detects phosphorylation at threonine 37 and 46) and GAPDH (Proteintech, Rosemont, IL, USA; Cat# HRP-60004; 1:5000 dilution). Secondary antibodies (anti-rabbit or anti-mouse IgG HRP-conjugated; Cell Signaling Technology) were used at 1:5000 dilution.

Protein bands were visualised using the SuperSignal West Pico Chemiluminescent Substrate (Thermo Fisher Scientific; Cat#34580), and were analysed using Gel Image System ver. 4.00 software (Tanon, China), while the Gel-Pro Analyzer was utilised to quantify the grayscale for protein analysis. GAPDH served as the internal control. The formula for calculating the mean grey value was as follows: Mean grey value = object value / internal control value. For phosphorylated proteins (e.g. p-AKT, p-mTOR, p-4EBP1), the ratios of phosphorylated to total protein levels were calculated to account for variations in total protein loading.

### 3-(4,5-Dimethylthiazol-2-yl)-2,5-diphenyltetrazolium bromide (MTT) assay

The MTT assay was conducted to measure the proliferation activity according to the manufacturers’ recommendation [31]. Briefly, a total of 5,000 cells per well in 96-well plates were incubated in 100-μL medium containing 0.5% FBS for 48 h with indicated concentrations of drugs. Cells cultured in a complete medium were utilised as controls. Following 48 h of incubation, the medium was aspirated, and the cells were washed three times with PBS (phosphate-buffered saline). Subsequently, 100 μL of MTT solution (5 mg/mL) was added to each well, and the plates were read using BioTek Synergy H1 Hybrid Multi-Mode Reader (Agilent Technologies, USA) at an absorbance of 490 nm.

### Reverse transcription-quantitative polymerase chain reaction

Reverse transcription-quantitative polymerase chain reaction (RT-qPCR) was employed to the expression level of HER2 expression in GES-1 as well as in three GC cell lines. The total RNA was extracted by the TRIzol method (Thermo Fisher Scientific, Waltham, MA, USA; Cat#15596026) and subsequently reverse transcribed into cDNA using the One-Step RNA PCR Kit (Takara Bio Inc., Shiga, Japan; Cat#RR024A) according to the manufacturers’ instructions. The RT-qPCR was performed using Applied Biosystems QuantStudio 5 Real-Time PCR System (Thermo Fisher Scientific, USA), and RT-qPCR conditions were as follows: an initial cycle at 95°C for 30 s and 40 cycles of 95°C for 5 s and 60°C for 30 s. Polymerase Chain Reaction (PCR) amplification was performed with specific primers of target genes. Cycles of amplification were 35–40. GAPDH were employed as housekeeping genes. Relative expression of genes was calculated by the 2^−ΔΔ^*^C^*^T^ method [32]. Primer sequences for *ERBB2* (gene symbol for HER2)*, SMO*, and *GAPDH* were designed using Primer-BLAST (NCBI) and validated for specificity and efficiency. The sequences of *ERBB2*, *SMO*, and *GAPDH* forward and reverse primers are shown below:

*ERBB2*: forward, 5’ TGCAGGGAAACCTGGAACTC-3’ and reverse, 5’ ACAGGGGTGGTATTGTTCAGC-3’;

*SMO:* forward, 5’ TCGAATCGCTACCCTGCTG-3’ and reverse, 5’ CAAGCCTCATGGTGCCATCT-3’.

*GAPDH:* forward, 5’GGAGCGAGATCCCTCCAAAAT-3’ and reverse, 5’ GGCTGTTGTCATACTTCTCATGG -3’.

### Drug combination analysis

Drug combination analysis was employed as previously described by Chou et al. [33]. For two-drug combination experiments, HGC27 cells were treated with different concentrations of Vis (MedChemExpress, Monmouth Junction, NJ, USA; Cat# HY-10255) or Cyc (Selleck Chemicals, Houston, TX, USA; Cat#S1146), as single agents as well as in combination with trastuzumab (Roche, Basel, Switzerland; purchased from the Second Hospital of Hebei Medical University Pharmacy). The dose-effect parameters of each drug alone, as well as in combination, were calculated to determine the combination index (CI) value. The combination index (CI)-isobologram equation, enabling a quantitative assessment of drug interactions, assigns values of CI < 1, = 1, and > 1 to indicate synergism, an additive effect, and antagonism, respectively. The CI was generated using the CompuSyn software (ComboSyn, Inc., Paramus, NJ, USA; version 1.0).

### Construction of the in vivo xenograft model

BALB/c nude mice (6 weeks old, male, body weight 20–25 g) were purchased from Model Animal Research Center of Hebei Medical University (Hebei, China; License No. SCXK 2023-0004). Mice were fed a standard diet containing 6% fat (LabDiet 5K52 formulation). For the *in vivo* tumour formation assays, 5 × 10^6^ HGC-27 cells in 100 μl of PBS were injected into both flanks of anesthetised mice. When the tumour volumes reached an average of 100 mm^3, the mice were randomly allocated into six groups, each consisting of five mice. The experimental groups were set up as follows: the control group: in which mice were intraperitoneally injected with PBS; the trastuzumab group: in which mice were intraperitoneally injected with trastuzumab, and the treatment was administered at a dose of 15 mg/kg three times per week for a duration of 2 weeks; the trastuzumab plus Vis combination therapy group: in which mice were intraperitoneally injected with 15 mg/kg trastuzumab for 3 consecutive days and 2 mg/kg Vis once daily for 2 weeks; the trastuzumab plus Cyc combination therapy group: in which mice were intraperitoneally injected with 15 mg/kg trastuzumab for 3 consecutive days and 2 mg/kg Cyc once daily for 2 weeks; and two SMO overexpression groups, in which tumour cells were transfected with 100 ug SMO overexpression plasmids for 48 h before inoculation. For anaesthesia, the mice were anesthetised with a mixture of ketamine (100 mg/kg) and xylazine (20 mg/mL) administered by intraperitoneal injection. At the end of the experiments, all mice were euthanised by cervical dislocation, and images were taken before measuring tumour weights. The width (W) and length (L) of the tumour were measured using a digital vernier caliper followed by determination of the tumour volume (V) — Formula: V (cm^3^) = (L × W^2^)/2. The animal use protocol underwent review and received approval from the Institutional Animal Care and Use Committee of Hebei Medical University (Approval No. 2024-AE205).

### Statistical analysis

All experiments were replicated three times, each with nine independent experimental runs performed. For comparisons, U-Mann-Whitney test and non-parametric Analysis of Variance (ANOVA) test (Kruskal-Wallis test), followed by Dunn’s post hoc test, was utilised. Differences were considered significant when *P* < 0.05. All data analyses were performed using GraphPad Prism 8.0 software (GraphPad Software, Inc., San Diego, CA, USA). The correlation between the HER2 and SMO expression and the tumour growth was calculated using the Spearman rank correlation analysis.

## Results

### HGC-27 cell line is sensitive to trastuzumab treatment and expresses high HER2 level

**Figure 1:**
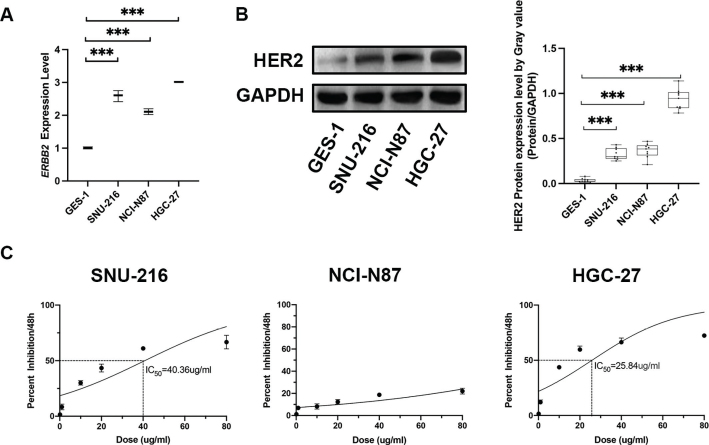
Effects of trastuzumab on the cell growth and detection of HER2 expression level in different GC cell lines. (a) The mRNA expression level of *ERBB2* in in normal gastric epithelial cell line GES-1 and in aforementioned three GC cells quantified using RT-qPCR. Data are presented as median and interquartile range (IQR) of three independent experiments. ***P < 0.001. (b) The protein level of HER-2 was measured by Western blot. (c) Cell viability of SNU-216, NCI-N87, and HGC-27 was detected by MTT assays after trastuzumab (0.1/1/10/20/40/80 ug/mL) treatment for 48 h.

The expression profiles of *ERBB2* in normal gastric epithelial cells GES-1 as well as in GC cell lines were validated using RT-qPCR. As shown in Figure 1a, the *ERBB2* expression is notably elevated in all GC cell lines, among which, HGC-27 showed the highest level of *ERBB2* expression (Tables 1 and 2, Supplementary Table 1). The Western blot analysis demonstrated that HGC-27 displayed a strongly enhanced expression of HER2 (Figure 1b). The MTT assay was further conducted to elucidate the trastuzumab sensitivity in GC cell lines. The concentration–response curves are drawn in Figure 1c. The viability of HGC-27 and SNU-216 cells was significantly reduced by trastuzumab in a dose-dependent manner, with an IC50 was 25.84 ug/mL and 40.36 ug/mL, respectively. Taken together, our data showed that HER2 expression was elevated in the GC cell lines. HGC-27 was chosen for subsequent analysis for its highest expression of HER2 at both mRNA and protein levels and was identified as the most sensitive to HER2 monoclonal antibody trastuzumab therapeutics.

**Table 1:** The description of statistical results of Kruskal-Wallis test.

Figures	Description	*p*	*H*	*df*
**1A**	HER2 expression level	0.045	5.44	3
**2C**	EdU+ cells%	0.024	3.71	2
**2D**	Relative colony number	0.021	6.34	2
**2F**	Tumour weight (g)	0.013	5.12	2
**2G**	Tumour volume (cm^2^)	0.009	8.26	2
**4B**	EdU+ cells%	<0.001	15.25	4
**4D**	Relative colony number	<0.001	11.42	4
**5A**	Tumour weight (g)	0.006	13.56	5
**5B**	Tumour volume (cm^2^)	<0.001	12.33	5
**6B**	AKT phosphorylation%	<0.001	15.20	5
**6C**	mTOR phosphorylation%	0.002	10.29	5
**6D**	4EBP1 phosphorylation%	0.005	11.93	5
**S2**	SMO expression level	<0.001	6.18	2

*p*: significance level; H: test value; df: degrees of freedom.

**Table 2:** The descriptive statistics of Median and Quartiles (Q1, Q3) for each experimental group.

Figures	Description	Groups	Median	Quartiles (Q1, Q3)
**1A**	HER2 expression level	GES-1	0.995	0.989, 1.002
		SNU-216	2.854	2.756, 3.001
		NCI-N87	1.997	1.902, 2.242
		HGC-27	3.084	3.084, 3.352
**2C**	EdU+ cells%	NC	26.631	24.553, 29.002
		+Vis	14.852	14.004, 16.292
		+Cyc	13.008	12.181, 14.017
**2D**	Relative Colony Number	NC	1.004	0.993, 1.020
		+Vis	0.457	0.420, 0.490
		+Cyc	0.417	0.398, 0.452
**2F**	Tumour weight (g)	NC	1.903	1.872, 1.940
		+Vis	1.497	1.430, 1.563
		+Cyc	1.410	1.392, 1.437
**2G**	Tumour volume (cm^2^)	NC	0.647	0.601, 0.687
		+Vis	0.323	0.301, 0.342
		+Cyc	0.303	0.289, 0.3103
**4B**	EdU+ cells%	+trastuzumab	21.391	20.352, 22.587
		+trastuzumab+Vis	12.801	12.238, 13.251
		+trastuzumab+Vis+SMO OE	22.836	22.100, 23.288
		+trastuzumab+Cyc	12.601	11.352, 14.196
		+trastuzumab+Cyc +SMO OE	20.802	19.028, 22.025
**4D**	Relative colony number	+trastuzumab	0.802	0.751, 0.828
		+trastuzumab+Vis	0.446	0.422, 0.462
		+trastuzumab+Vis+SMO OE	0.831	0.794, 0.861
		+trastuzumab+Cyc	0.324	0.290, 0.361
		+trastuzumab+Cyc +SMO OE	0.906	0.893, 0.924
**5A**	Tumour weight (g)	NC	2.197	2.150, 2.224
		+trastuzumab	1.717	1.652, 1.801
		+trastuzumab+Vis	1.347	1.302, 1.380
		+trastuzumab+Vis+SMO OE	1.724	1,732, 1.760
		+trastuzumab+Cyc	1.262	1.225, 1.352
		+trastuzumab+Cyc +SMO OE	1.632	1.600, 1.653
**5B**	Tumour volume (cm^2^)	NC	0.690	0.651, 0.726
		+trastuzumab	0.574	0.549, 0.603
		+trastuzumab+Vis	0.221	0.202, 0.237
		+trastuzumab+Vis+SMO OE	0.584	0.521, 0.629
		+trastuzumab+Cyc	0.265	0.248, 0.283
		+trastuzumab+Cyc +SMO OE	0.518	0.513, 0.523
**6B**	AKT phosphorylation%	NC	0.997	0.984, 1.004
		+trastuzumab	0.771	0.748, 0.802
		+trastuzumab+Vis	0.301	0.281, 0.320
		+trastuzumab+Vis+SMO OE	1.072	1.028, 1.134
		+trastuzumab+Cyc	0.344	0.301, 0.401
		+trastuzumab+Cyc+SMO OE	0.847	0.784, 0.893
**6C**	mTOR phosphorylation%	NC	0.998	0.993, 1.002
		+trastuzumab	0.777	0.784, 0.802
		+trastuzumab+Vis	0.332	0.301, 0.395
		+trastuzumab+Vis+SMO OE	1.098	1.054, 1.134
		+trastuzumab+Cyc	0.611	0.582, 0.621
		+trastuzumab+Cyc+SMO OE	0.903	0.884, 0.934
**6D**	4EBP1 phosphorylation%	NC	0.994	0.985, 1.004
		+trastuzumab	0.619	0.602, 0.635
		+trastuzumab+Vis	0.296	0.287, 0.302
		+trastuzumab+Vis+SMO OE	0.802	0.782, 0.823
		+trastuzumab+Cyc	0.457	0.441, 0.481
		+trastuzumab+Cyc+SMO OE	0.892	0.884, 0.901

### Hh inhibitors Vis and Cyc suppress the cell proliferation and promote apoptosis in vitro and in vivo

**Figure 2:**
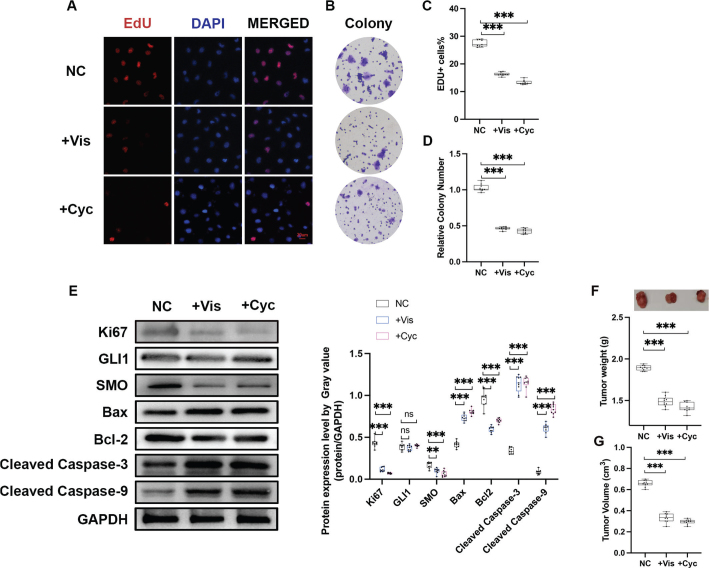
Effects of Cyc and Vis on proliferation and apoptosis of HGC-27. EdU assays (a and c) and colony formation assays (b and d) were performed to evaluate the effects of Vis (50 nM) and Cyc (10 nM) on the cell proliferation of HGC-27 cells *in vitro*. Data are presented as median and IQR of three independent experiments. ***P < 0.001. (e) The protein expressions of Hh components were validated using Western blot analysis. (f) Effects of Vis and Cyc on the cell proliferation *in vivo*. The top panel shows the tumour xenografts. The lower panel represents the quantified tumour weight. Data are presented as median and IQR, and *n* = 5 mice in each group. ***P < 0.001. (g) Tumour volume of nude mice. Data are presented as median and IQR, and *n* = 5 mice in each group. ***P < 0.001. The tumour length (tumour long diameter), tumour width, and maximum volume of all tumours per mouse were measured and recorded in Table 3 and Supplementary Table 2. Abbreviations: Vis: vismodegib; Cyc, cyclopamine.

Next, we investigated the effects of two classic Hh inhibitors Vis and Cyc on the proliferation, apoptosis, migration, and invasion of HGC-27 cells. In line with previous studies, administration of 50 nM Vis or 10 nM Cyc for 72 h notably decreased EdU positive cells as well as colony formation capacity, compared to PBS-treated control (Figure 2a–d, Tables 1 and 2, Supplementary Table 1) [34]. In addition, the administration of neither Vis nor Cyc altered the migration or invasion capacity of HGC-27 cells (Supplementary Figure 1). We further examined the expressions of Hh-related molecules at protein levels. As shown in Figure 2e, SMO, a core component of canonical Hh signalling, was significantly repressed after the administration of Vis or Cyc, while GLI1 remained intact. As expected, the protein level of the cell proliferation marker Ki67 was significantly downregulated after drug administration. The expression levels of apoptotic proteins were further detected. Both Vis and Cyc treatments significantly upregulated the expression of Bax, a pro-apoptotic protein belonging to the Bcl-2 protein family, in HGC-27 cells, while downregulating the expression of Bcl-2. Furthermore, apoptotic markers, including cleaved caspase-3 and cleaved caspase-9, were upregulated by Vis and Cyc administration. These findings suggest that Vis and Cyc can effectively inhibit cell growth and promote apoptosis in HGC-27 cells, accompanied by the downregulation of SMO expression. In an attempt to validate the inhibitory role of Vis and Cyc in GC, tumour xenografts in nude mice were applied to evaluate the effect of Vis and Cyc on tumour growth. As depicted in Figure 2f and g, 15 days post tumour cell transplantation, Vis (50 nM for 72 h) or Cyc (10 nM for 72 h) pretreated HGC-27 cells formed significantly smaller tumour mass and volume in nude mice, compared with the blank controls (Tables 1–3, Supplementary Table 1).

**Table 3:** The tumour length, tumour width, and maximum volume of two tumours in both flanks per mouse.

Groups	Replicates	Tumour length (right/left) (cm)	Tumour width (right/left) (cm)	Maximum volume (cm^3^)
**NC**	#1	1.71/1.68	0.89/0.86	1.300
	#2	1.64/1.69	0.95/0.84	1.336
	#3	1.65/1.74	0.90/0.81	1.239
	#4	1.65/1.69	0.92/0.88	1.353
	#5	1.62/1.71	0.96/0.85	1.364
**+Vis**	#1	1.10/0.99	0.76/0.70	0.560
	#2	1.42/1.40	0.67/0.65	0.614
	#3	1.57/1.54	0.62/0.66	0.627
	#4	1.53/0.63	0.63/0.66	0.630
	#5	1.62/1.59	0.63/0.62	0.627
**+Cyc**	#1	1.70/1.72	0.54/0.53	0.489
	#2	1.67/1.67	0.56/0.59	0.553
	#3	1.68/1.63	0.56/0.62	0.577
	#4	1.60/1.62	0.61/0.60	0.589
	#5	1.64/1.60	0.60/0.62	0.603

NC group: mice were intraperitoneally injected with HGC-27 cells; +Vis group: mice were intraperitoneally injected with HGC-27 cells pretreated with 50 nM vismodegib for 72 h. +Cyc group: mice were intraperitoneally injected with HGC-27 cells pretreated with 10 nM cyclopamine for 72 h.

### Vis and Cyc both show synergistic effects on repressing HGC-27 cell growth when combined with trastuzumab

**Figure 3:**
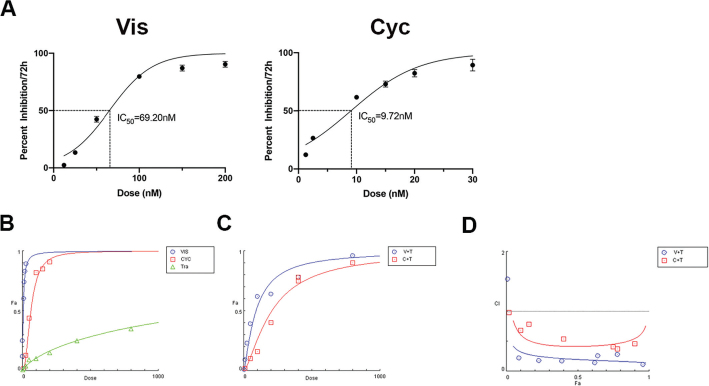
Graphical representation generated from the CompuSyn Report. (a) Cell viability of HGC-27 was detected by MTT assays after treatment with Vis (12.5–400 nM) or Cyc (1.25–40 nM) for 72 h. (b) Dose-effect curve for each drug. (c) Dose-effect curve for combination therapy. (d) Combined index plot. Abbreviations: Tra: trastuzumab; V+T: the combination of vismodegib and trastuzumab; C+T: the combination of cyclopamine and trastuzumab.

Inspired by the above finding that two Hh inhibitors Cyc and Vis exerted anti-proliferation effects in HER2-positive GC cells, we further investigated the synergistic effects of Hh inhibitors and trastuzumab in HGC-27 cell line. Dose-concentration effects of Cyc and Vis on HGC-27 cells were determined via MTT assay. The initial concentration was set as 1.25 nM and 12.5nM for Cyc and Vis, respectively, according to published literature [35]. The drugs were subsequently diluted to 6 concentrations with two-fold serial dilutions to establish the IC50 value. The IC50 values for Cyc and Vis were 9.72 nM and 69.20 nM, respectively (Figure 3a) [36]. As shown in Figure 3b and c, the Dose-effect parameters of each drug alone, as well as in combination, were calculated to determine the CI value. The combination therapy for Cyc and trastuzumab was carried out at a ratio of 1:100. As a result, Cyc and trastuzumab played a synergistic role, with CI = 0.414 when Fa = 0.5. Likewise, the combination of Vis and trastuzumab also exerts a strong synergistic effect, with the CI value identified as 0.197 (Figure 3d and Table 4). Overall, our results demonstrated that Hh inhibitors and HER2 antibody trastuzumab exhibited a strong synergistic relationship in HGC-27 cells.

**Table 4:** Assessment of combinatorial effect of two Hh inhibitors and trastuzumab in HGC-27 cell line.

CI	Tra+Vis	Tra+Cyc
ED_50_	0.19713	0.41426
ED_75_	0.17030	0.45288
ED_90_	0.15417	0.54729
ED_95_	0.14678	0.64072
ED_97_	0.14266	0.72216
Conclusion	Synergism	Synergism

CI data was determined by the CompuSyn soft, CI < 1, CI > 1 and CI = 1 indicate synergism, antagonism and additivity, respectively. ED: Maximum reaction intensity dosage; Tra: trastuzumab.

### Vis and Cyc suppress cell growth and promote apoptosis in an SMO-dependent manner

**Figure 4:**
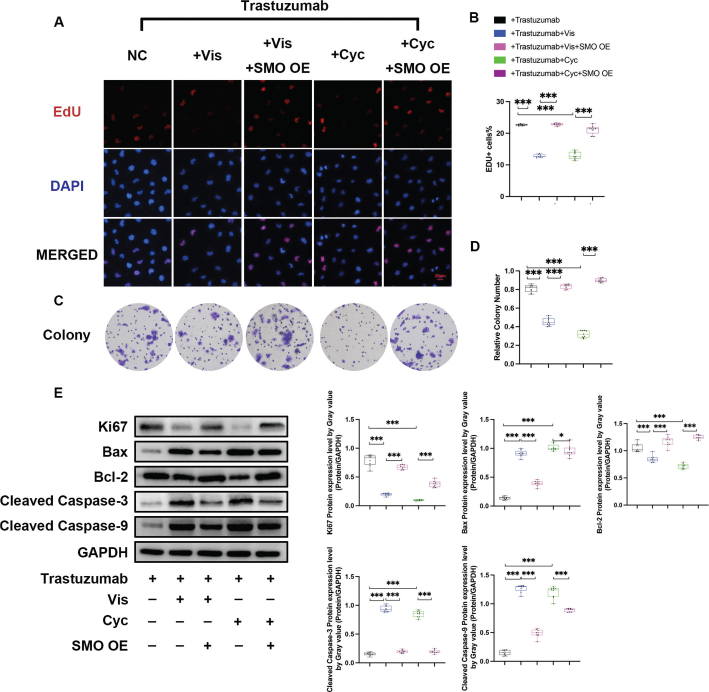
Effects of Hh inhibitors as well as SMO overexpression on the cell proliferation and apoptosis in HGC-27 cells. (a–d) Effects of Vis (50 nM) and Cyc (10 nM) on the proliferation capacity of HGC-27 cells in the presence of trastuzumab, as demonstrated by EdU assay (a and b) and colony formation assay (c and d). Data are presented as median and IQR of three independent experiments. ***P < 0.001. (e) The protein levels of proliferation marker as well as apoptotic markers in five experimental groups was assessed using Western blot analysis. Abbreviations: SMO OE, SMO overexpression.

In line with MTT results, the addition of 50 nM or 10 nM Cyc exerted significantly stronger growth inhibition as well as apoptosis promotion on HGC-27 cells than trastuzumab alone, as demonstrated by less EdU positive cells, less colony formation, and a significantly smaller tumour mass. Western blot was conducted on the related indicators of proliferation and apoptosis of HGC-27 cells. It was suggested that the addition of Hh inhibitor could significantly increase Bax, cleaved caspase-3 and cleaved caspase-9, and decrease Bcl-2, with statistical significance, when compared with trastuzumab-only therapy. Since the expression of SMO was notably restrained after Cyc and Vis administration, we next overexpressed SMO to explore whether its downregulation was essential for the anti-growth and pro-apoptotic effects observed before. The *SMO* expression level was significantly elevated in cells transfected with SMO overexpression vector (Supplementary Figure 2). Our results demonstrated that transfection of SMO plasmids (SMO OE) enhanced EdU-positive cell count, colony formation, and Ki67 protein levels in HGC-27 cells, while significantly suppressing apoptosis indicators. These findings suggest that the SMO depletion was crucial to the anti-growth and pro-apoptotic capacity of Hh inhibitors (Figure 4a–e, Tables 1 and 2, Supplementary Table 1).

**Figure 5:**
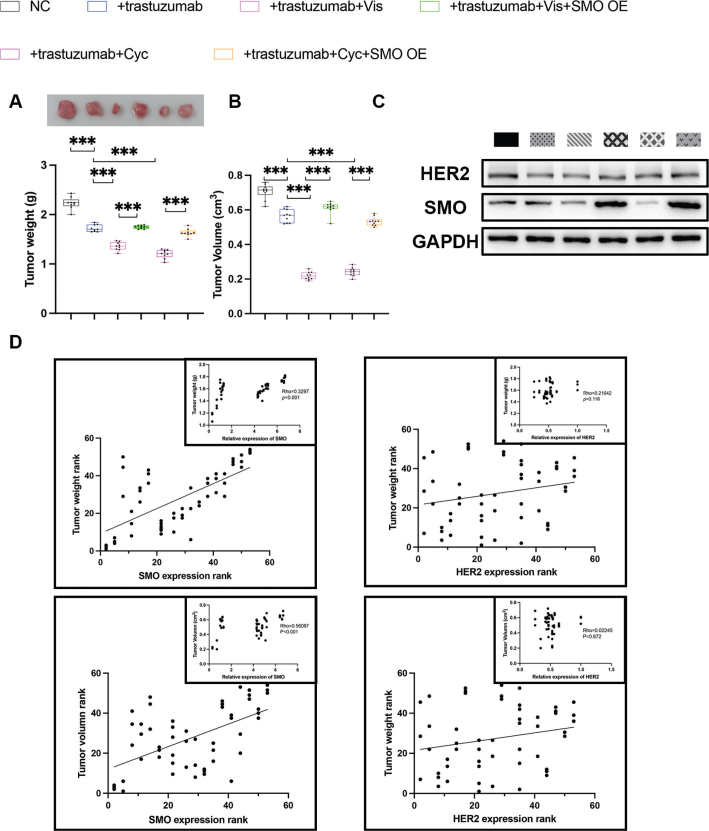
Correlation between the HER2 and SMO expression and the tumour growth. (a) Two weeks post the initial injection, HGC-27 xenografts in six different experimental groups were harvested and weighed. Mice were treated with trastuzumab (15 mg/kg), Vis (2 mg/kg), or Cyc (2 mg/kg). The top panel shows the tumour xenografts. The lower panel represents the quantified tumour weight. Data are presented as median and IQR, and *n* = 5 mice in each group. ***P < 0.001. (b) Tumour volume of nude mice. Data are presented as median and IQR, and *n* = 5 mice in each group. ***P < 0.001; The tumour length (tumour long diameter), tumour width, and maximum volume of all tumours per mouse were measured and recorded in Table 5, Supplementary Table 1 and Supplementary Table 3. (c) The protein levels of SMO and HER2 in six different experimental groups. (d) The relationships between SMO/HER2 expression level and tumour weight and tumour size were analysed using Spearman’s Rho Rank Correlation test. For each of the four relationships, two scatter diagrams including one large plot on the ranked data and one small plot (in the upper right corner of the large plot) on the real data was generated. ***P < 0.001.

To evaluate the effects of SMO expression *in vivo*, HGC-27 cells were inoculated s.c. into nude mice. As demonstrated in Figure 5a and b, tumours treated with trastuzumab were significantly smaller than those not treated with trastuzumab. The tumour growth was further inhibited in the trastuzumab+Vis and trastuzumab+Cyc groups compared with the trastuzumab group. As predicted by the *in vitro* studies, we found that SMO overexpression led to a significantly greater tumour weight and volume than trastuzumab+Vis and trastuzumab+Cyc groups (Tables 1, 2 and 5, Supplementary Table 1). We detected the protein level of HER2 and SMO to analyse the correlation between the HER2 and SMO expression, and the tumour growth (Figure 5c and d, Tables 1, 2 and 6, Supplementary Table 1) [37]. Both tumour weight and tumour volume showed significantly moderate correlations (*r* = 0.3297) and strong correlation (*r* = 0.5609), respectively, with SMO expression, while HER2 expression level exhibited no obvious correlation with tumour weight or tumour volume.

**Table 5:** The tumour length, tumour width, and maximum volume of two tumours in both flanks per mouse.

Groups	Replicates	Tumour length (right/left) (cm)	Tumour width (right/left) (cm)	Maximum volume (cm^3^)
**NC**	#1	1.76/1.72	0.88/0.92	1.409
	#2	1.70/1.71	0.88/0.87	1.305
	#3	1.76/1.76	0.89/0.90	1.410
	#4	1.81/1.80	0.87/0.83	1.305
	#5	1.77/1.82	0.88/0.85	1.343
**+trastuzumab**	#1	1.70/1.66	0.85/0.84	1.200
	#2	1.68/1.65	0.84/0.82	1.147
	#3	1.67/1.65	0.83/0.81	1.117
	#4	1.64/1.60	0.83/0.85	1.143
	#5	1.62/1.67	0.84/0.80	1.106
**+trastuzumab+Vis**	#1	1.50/1.49	0.56/0.56	0.469
	#2	1.42/1.54	0.58/0.63	0.544
	#3	1.47/1.57	0.62/0.61	0.574
	#4	1.51/1.55	0.68/0.58	0.610
	#5	1.58/1.63	0.56/0.55	0.494
**+trastuzumab+Vis+SMO OE**	#1	1.77/1.72	0.85/0.83	1.232
	#2	1.82/1.78	0.78/0.80	1.123
	#3	1.81/1.78	0.81/0.78	1.135
	#4	1.73/1.74	0.85/0.82	1.210
	#5	1.76/1.84	0.86/0.76	1.182
**+trastuzumab+Cyc**	#1	1.43/1.55	0.65/0.65	0.630
	#2	1.40/1.52	0.68/0.61	0.606
	#3	1.41/1.49	0.62/0.63	0.557
	#4	1.52/1.52	0.67/0.69	0.703
	#5	1.51/1.46	0.66/0.65	0.637
**+trastuzumab+Cyc+SMO OE**	#1	1.72/1.73	0.85/0.83	1.217
	#2	1.82/1.73	0.77/0.84	1.150
	#3	1.84/1.78	0.81/0.80	1.173
	#4	1.73/1.75	0.84/0.83	1.213
	#5	1.76/1.86	0.81/0.76	1.114

The control group: mice were intraperitoneally injected with PBS; the trastuzumab group: mice were intraperitoneally injected with trastuzumab (15 mg/kg for three times) for 2 weeks; the trastuzumab plus Vis combination therapy group: mice were intraperitoneally injected with trastuzumab (15 mg/kg for three times) and Vis (2 mg/kg once daily) for 2 weeks; the trastuzumab plus Cyc combination therapy group: mice were intraperitoneally injected with trastuzumab (15 mg/kg for three times) and Cyc (2 mg/kg once daily) for 2 weeks; and two SMO overexpression groups: tumour cells were transfected with 100 ug SMO overexpression plasmids for 48 h before inoculation.

**Table 6:** Spearman’s rank correlation between the HER2 and SMO expression and the tumour growth.

Variables	*p*	*t* (*N*-2)	Rho
Tumour weight, SMO expression	<0.001	7.101	0.33
Tumour volume, SMO expression	<0.001	9.324	0.56
Tumour weight, HER2 expression	0.116	0.824	0.22
Tumour volume, HER2 expression	0.872	0.303	0.02

### Vis and Cyc exert anti-tumour effects by mediating the AKT/mTOR/4EBP1 signalling

**Figure 6:**
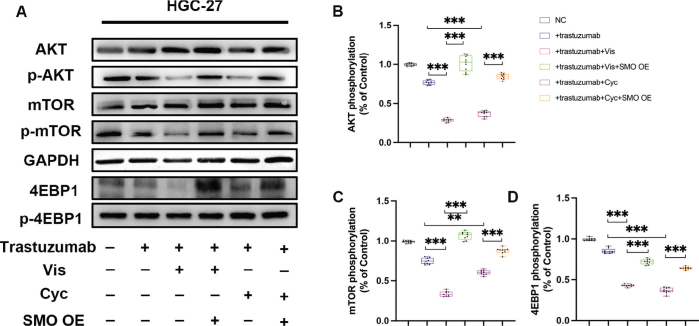
The activity of AKT/mTOR signalling in HGC-27 cells was measured by Western blot analysis. (a) Western blot analysis of AKT/mTOR signalling in HGC-27 cells treated with Vis (50 nM) or Cyc (10 nM). (b) Relative AKT phosphorylation levels in HGC-27 cells were determined by western blot analysis with total AKT as the loading control for each sample. (c) Relative mTOR phosphorylation levels in HGC-27 cells were determined by western blot analysis with total mTOR as the loading control for each sample. (d) Relative 4EBP1 phosphorylation levels in HGC-27 cells were determined by western blot analysis with total 4EBP1 as the loading control for each sample. Data are presented as median and IQR of three independent experiments. **P < 0.01, ***P < 0.001.

Previous studies have indicated that AKT/mTOR/4EBP1 signalling pathway was amplified in GC, and was involved in abnormally enhanced cell growth of GC cells and accumulating evidence has revealed the crosstalk between Hh and mTOR/4EBP1 signalling [38, 39]. Thus, we further assessed the AKT/mTOR/4EBP1 activity in HGC-27 cells. As exhibited in Figure 6a–c, the combination therapy significantly suppressed the activation of AKT/mTOR/4EBP1 signalling when compared with trastuzumab only administration (Tables 1 and 2, Supplementary Table 1). Meanwhile, we found that the compromised proliferation capacity was completely rescued by plasmids-mediated SMO overexpression, suggesting that SMO downregulation is essential for Vis or Cyc to exert their anti-growth effects on HGC-27 cells.

## Discussion

Although important progress has been made regarding the prevention and treatment for GC, therapeutic options, especially for patients in their advanced stages, are still limited. The standard treatment for inoperable GC is still systemic chemotherapy [40]. Benefiting from the development of targeted cancer therapeutics, the HER receptors have been chosen as putative molecular targets in GC treatment. The approval of the monoclonal HER2-targeted antibody trastuzumab showed significant improvements in clinical outcomes for the treatment of advanced or metastatic GC [15, 41, 42]. Essentially, before initiating any systemic chemotherapy, patients are tested for expression of the HER2, and people with IHC 2+ with ISH+ or IHC 3+ are introduced to receive a combination of a platinum/fluoropyrimidine-based chemotherapy and trastuzumab [43].

However, the use of trastuzumab remains limited due to insufficient cellular sensitivity and the development of acquired drug resistance. Combinatorial strategies building on the chemotherapy/trastuzumab backbone, with the addition of immune checkpoint inhibitors or other targeted therapies, carry hope for improving patient outcomes. For instance, Shi and his colleagues recently reported that silencing the HER2-SHCBP1-PLK1 axis promoted tumour growth and restored the trastuzumab sensitivity to GC [44]. With the expanding number of available drugs, it is important to establish clear guidelines for their effective use in clinical settings [45]. Current evidence suggests that combination therapies involving Hh inhibitors (e.g. Vis) and trastuzumab may delay or mitigate resistance mechanisms, but long-term resistance data remain limited. In preclinical studies, combining Vis with trastuzumab reduced tumour growth and metastasis in HER2-positive trastuzumab-resistant breast cancer models (e.g. BT474-TtzmR cells). This combination also inhibited cancer stem cells, which are often implicated in therapeutic resistance [46]. Meanwhile, emerging preclinical studies highlight the potential of Vis to reverse trastuzumab resistance through SMO-dependent and independent mechanisms. For instance, in HER2-positive GC, SMO inhibition reduced tumour growth and restored trastuzumab sensitivity by inactivating the AKT/mTOR pathway [46]. In our current work, we elaborate on the potential mechanism underlying the regulatory effects of Hh inhibitors in GC development and offer a promising therapeutic strategy for HER2-positive GC.

In GC, accumulating studies have provided that AKT/mTOR activation is often observed in clinical specimens as well as in GC cell lines and the phosphorylation level of mTOR is positively correlated with cancer development, chemoresistance, and poor clinical outcome [47, 48]. Given its prominent role in GC development, a number of molecules targeting the AKT/mTOR pathway have been developed and have been proven to play a reliable role in retraining tumorigenesis, progression, and metastasis [49–51]. In HER2-positive GC, trastuzumab binds HER2, blocking its dimerization and subsequent PI3K/AKT/mTOR activation. This inhibition reduces phosphorylation of AKT and mTOR, downregulating effector molecules such as 4EBP1, ultimately suppressing protein synthesis and tumour growth [52]. Also, our study reveals crosstalk between HER2 and SMO: Combining trastuzumab with Hh inhibitors (Vis or Cyc) synergistically suppresses SMO, further inactivating AKT/mTOR signalling and enhancing anti-tumour effects. This dual targeting strategy addresses compensatory signalling mechanisms, overcoming resistance to single-agent therapy.

It is worth mentioning that GLI1 could also be activated by AKT and ERK in an SMO-Independent manner. Emerging literature demonstrates that AKT and ERK signalling pathways can directly phosphorylate and stabilise GLI1, bypassing canonical SMO-dependent activation. This mechanism is particularly relevant in cancers where SMO inhibitors (e.g. vismodegib, sonidegib) fail to suppress tumour growth due to intrinsic or acquired resistance. For instance, it has been reported that AKT phosphorylates GLI1 at specific residues (e.g. Ser84), enhancing its nuclear translocation, transcriptional activity, and resistance to proteasomal degradation. In pancreatic and breast cancers, AKT-driven GLI1 activation promotes tumour stemness and chemoresistance, even in the presence of SMO inhibitors [20, 53]. The SMO-independent activation of GLI1 represents a major therapeutic challenge. For example, in basal cell carcinoma, nuclear localisation of SMO via a nuclear/nucleolar localisation signal activates GLI1 independently of canonical SMO inhibitors, explaining resistance in ~80% of patients [54]. These escape mechanisms highlight the need for combination therapies targeting both upstream (SMO) and downstream (AKT/ERK/GLI1) components of the pathway.

Taken together, our current work unveiled that Hh inhibitors and anti-HER2 antibody trastuzumab can make an effective combination for the treatment for HER2-positive GC patients. The anti-tumour effects of trastuzumab can be amplified when combined with Hh inhibitors Vis and Cyc. There remains a critical need for the development of more effective agents. Future studies are urgently needed to detect novel therapeutic molecular and predictive factors in order to provide better and optimal treatment modalities for GC treatment.

## Limitations

The study shows promising results in HGC-27 cell lines and animal models, the translation of these findings to clinical practice may however, face challenges. The effectiveness observed *in vitro* and *in vivo* were not examined in human patients with HER2-positive GC. The study primarily focusses on the HER2-positive HGC-27 cell line, which may not fully represent the heterogeneity of HER2-positive GC. The generalisability of the findings to a broader patient population is unclear. Future studies using patient-derived xenograft models should be performed to enhance the clinical relevance and translatability of our findings. In addition, future studies should include GLI inhibitors (e.g. GANT61) to confirm SMO dependency and address potential non-canonical Hh signalling. Taken together, while the current study presents promising findings regarding the potential use of Hh inhibitors in combination with HER2-targeted trastuzumab for treating HER2-positive GC, several limitations, including lacing comprehensive clinical data and limited generalisation, must be considered when interpreting the results and planning future research or clinical trials.

## Conclusions

To conclude, this study underscores the potential of Hh inhibitors, Vis and Cyc, to enhance trastuzumab’s anti-tumour activity via SMO-regression and AKT/mTOR pathway inactivation. This suggests a promising approach of Hh inhibition combined with HER2-targeted trastuzumab for HER2-positive GC treatment.

## Supplementary Material

Hedgehog inhibitors exert anti-proliferation effects and synergistically interact with trastuzumab in HER2-positive gastric cancer models

## Data Availability

The data used to support the findings of this study are available from the corresponding author upon request.
