# Supplementary material for: Hedgehog inhibitors exert anti-proliferation effects and synergistically interact with trastuzumab in HER2-positive gastric cancer models
Source: Acta Oncol. 2025 May 27;64:42219. doi: 10.2340/1651-226X.2025.42219 (PMC12131914; doi:10.2340/1651-226X.2025.42219)
Supplement: Hedgehog inhibitors exert anti-proliferation effects and synergistically interact with trastuzumab in HER2-positive gastric cancer models [file AO-64-42219-s1.pdf]

Supplementary material has been published as submitted. It has not been copyedited, or typeset by Acta Oncologica

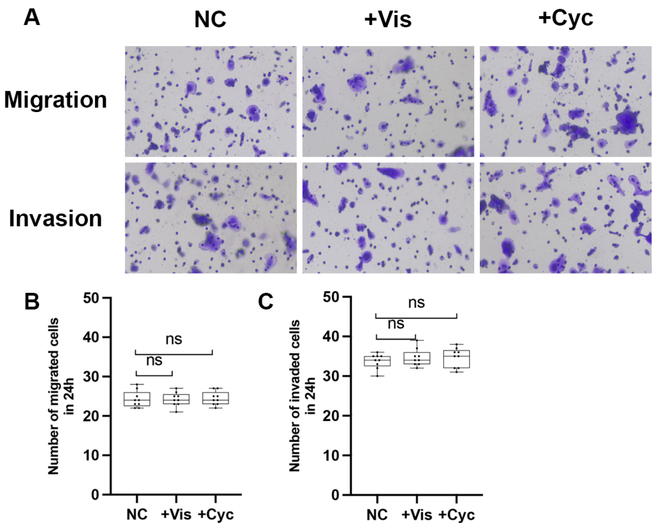

Figure S1 Administration of Vis or Cyc did not alter the migration or invasion of HGC-27 cells.

After 72h exposure of 50nM Vis or 10nM Cyc, migration assay and invasion assay (A) were performed. Experiments were performed in triplicates. ns: not statistically significant (B and C).

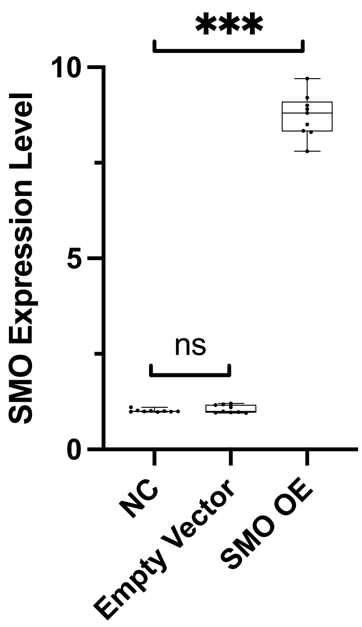

Figure S2 The SMO expression level was significantly elevated in cells transfected with SMO overexpression vector.

HGC-27 GC cells were transfected with the SMO overexpression vector or empty vector. The mRNA expression of SMO was significantly increased in the SMO OE group compared with that in the Empty vector and control groups, ns: not statistically significant, ***P<0.001.

Table S1 The description of Dunn's post hoc Test.

Figures	Description	Comparison	*p*
**1A**	HER2 expression level	GES-1 vs. SNU-216	< 0.001
		GES-1 vs. NCI-N87	0.031
		GES-1 vs. HGC-27	< 0.001
		SNU-216 vs. NCI-N87	< 0.001
		SNU-216 vs. HGC-27	0.043
**2C**	EdU+ cells%	NC vs. +Vis	0.002
		NC vs. +Cyc	0.001
		+Vis vs. +Cyc	0.023
**2D**	Relative colony number	NC vs. +Vis	0.021
		NC vs. +Cyc	0.015
		+Vis vs. +Cyc	0.106
**2F**	Tumor weight (g)	NC vs. +Vis	0.030
		NC vs. +Cyc	0.033
		+Vis vs. +Cyc	0.028
**2G**	Tumor volume (cm^2)	NC vs. +Vis	0.019
		NC vs. +Cyc	0.013
		+Vis vs. +Cyc	0.094
**4B**	EdU+ cells%	+trastuzumab vs. +trastuzumab+Vis	< 0.001
		+trastuzumab vs. +trastuzumab+Cyc	< 0.001
		+trastuzumab+Vis vs. +trastuzumab+Vis+SMO OE	< 0.001
		+trastuzumab+Cyc vs. +trastuzumab+Cyc +SMO OE	0.001
		+trastuzumab vs. +trastuzumab+Vis+SMO OE	0.893
		+trastuzumab vs. +trastuzumab+Cyc +SMO OE	0.232
		+trastuzumab+Vis vs. +trastuzumab+Cyc	0.771
		+trastuzumab+Vis vs. +trastuzumab+Cyc +SMO OE	< 0.001
		+trastuzumab+Vis+SMO OE vs. + trastuzumab+Cyc	< 0.001
		+trastuzumab+Vis+SMO OE vs. +trastuzumab+Cyc +SMO OE	0.0432
**4D**	Relative colony	+trastuzumab vs. +trastuzumab+Vis	0.002

	number	+trastuzumab vs. +trastuzumab+Cyc	< 0.001
		+trastuzumab+Vis vs. +trastuzumab+Vis+SMO OE	0.013
		+trastuzumab+Cyc vs. +trastuzumab+Cyc +SMO OE	< 0.001
		+trastuzumab vs. +trastuzumab+Vis+SMO OE	0.845
		+trastuzumab vs. +trastuzumab+Cyc +SMO OE	0.332
		+trastuzumab+Vis vs. +trastuzumab+Cyc	< 0.001
		+trastuzumab+Vis vs. +trastuzumab+Cyc +SMO OE	< 0.001
		+trastuzumab+Vis+SMO OE vs. + trastuzumab+Cyc	< 0.001
		+trastuzumab+Vis+SMO OE vs. +trastuzumab+Cyc +SMO OE	0.014
**5A**	Tumor weight (g)	NC vs. +trastuzumab	0.002
		+trastuzumab vs. +trastuzumab+Vis	< 0.001
		+trastuzumab vs. +trastuzumab+Cyc	0.039
		+trastuzumab+Vis vs. +trastuzumab+Vis+SMO OE	0.025
		+trastuzumab+Cyc vs. +trastuzumab+Cyc +SMO OE	0.016
		+NC vs. +trastuzumab+Vis	< 0.001
		+NC vs. +trastuzumab+Vis+SMO OE	< 0.001
		+NC vs. +trastuzumab+Cyc	< 0.001
		+NC vs. +trastuzumab+Cyc +SMO OE	0.001
		+trastuzumab vs. +trastuzumab+Vis+SMO OE	0.674
		+trastuzumab vs. +trastuzumab+Cyc +SMO OE	0.602
		+trastuzumab+Vis vs. +trastuzumab+Cyc	0.098
		+trastuzumab+Vis vs. trastuzumab + Cyc + SMO OE	0.002
		+transtuzumab+Vis+SMO OE vs. +trastuzumab+Cyc	< 0.001
		+transtuzumab+Vis+SMO OE vs. +trastuzumab+Cyc +SMO OE	0.023
**5B**	Tumor volume (cm^2)	NC vs. +trastuzumab	< 0.001
		+trastuzumab vs. +trastuzumab+Vis	< 0.001
		+trastuzumab vs. +trastuzumab+Cyc	< 0.001
		+trastuzumab+Vis vs. +trastuzumab+Vis+SMO OE	0.005
		+trastuzumab+Cyc vs. +trastuzumab+Cyc +SMO OE	0.002
		+NC vs. +trastuzumab+Vis	< 0.001
		+NC vs. +trastuzumab+Vis+SMO OE	0.027

		+NC vs. +trastuzumab+Cyc	< 0.001
		+NC vs. +trastuzumab+Cyc +SMO OE	< 0.001
		+trastuzumab vs. +trastuzumab+Vis+SMO OE	0.021
		+trastuzumab vs. +trastuzumab+Cyc +SMO OE	0.142
		+trastuzumab+Vis vs. +trastuzumab+Cyc	0.062
		+trastuzumab+Vis vs. trastuzumab + Cyc + SMO OE	< 0.001
		+transtuzumab+Vis+SMO OE vs. +trastuzumab+Cyc	< 0.001
		+transtuzumab+Vis+SMO OE vs. +trastuzumab+Cyc+SMO OE	0.009
**6B**	AKT phosphorylation%	NC vs. +trastuzumab	0.032
		+trastuzumab vs. +trastuzumab+Vis	< 0.001
		+trastuzumab vs. +trastuzumab+Cyc	0.023
		+trastuzumab+Vis vs. +trastuzumab+Vis+SMO OE	0.014
		+trastuzumab+Cyc vs. +trastuzumab+Cyc +SMO OE	0.007
		+NC vs. +trastuzumab+Vis	< 0.001
		+NC vs. +trastuzumab+Vis+SMO OE	0.102
		+NC vs. +trastuzumab+Cyc	< 0.001
		+NC vs. +trastuzumab+Cyc +SMO OE	0.023
		+trastuzumab vs. +trastuzumab+Vis+SMO OE	< 0.001
		+trastuzumab vs. +trastuzumab+Cyc +SMO OE	0.028
		+trastuzumab+Vis vs. +trastuzumab+Cyc	0.015
		+trastuzumab+Vis vs. trastuzumab + Cyc + SMO OE	< 0.001
		+transtuzumab+Vis+SMO OE vs. +trastuzumab+Cyc	< 0.001
		+transtuzumab+Vis+SMO OE vs. +trastuzumab+Cyc+SMO OE	0.011
**6C**	mTOR phosphorylation%	NC vs. +trastuzumab	0.022
		+trastuzumab vs. +trastuzumab+Vis	0.002
		+trastuzumab vs. +trastuzumab+Cyc	0.013
		+trastuzumab+Vis vs. +trastuzumab+Vis+SMO OE	0.012
		+trastuzumab+Cyc vs. +trastuzumab+Cyc +SMO OE	0.020
		+NC vs. +trastuzumab+Vis	< 0.001
		+NC vs. +trastuzumab+Vis+SMO OE	0.034
		+NC vs. +trastuzumab+Cyc	< 0.001

		+NC vs. +trastuzumab+Cyc +SMO OE	0.076
		+trastuzumab vs. +trastuzumab+Vis+SMO OE	0.001
		+trastuzumab vs. +trastuzumab+Cyc +SMO OE	0.048
		+trastuzumab+Vis vs. +trastuzumab+Cyc	< 0.001
		+trastuzumab+Vis vs. trastuzumab + Cyc + SMO OE	< 0.001
		+transtuzumab+Vis+SMO OE vs. +trastuzumab+Cyc	< 0.001
		+transtuzumab+Vis+SMO OE vs. +trastuzumab+Cyc+SMO OE	0.001
**6D**	4EBP1 phosphorylation%	NC vs. +trastuzumab	0.021
		+trastuzumab vs. +trastuzumab+Vis	0.005
		+trastuzumab vs. +trastuzumab+Cyc	0.023
		+trastuzumab+Vis vs. +trastuzumab+Vis+SMO OE	0.003
		+trastuzumab+Cyc vs. +trastuzumab+Cyc +SMO OE	0.015
		+NC vs. +trastuzumab+Vis	< 0.001
		+NC vs. +trastuzumab+Vis+SMO OE	< 0.001
		+NC vs. +trastuzumab+Cyc	< 0.001
		+NC vs. +trastuzumab+Cyc +SMO OE	< 0.001
		+trastuzumab vs. +trastuzumab+Vis+SMO OE	0.002
		+trastuzumab vs. +trastuzumab+Cyc +SMO OE	< 0.001
		+trastuzumab+Vis vs. +trastuzumab+Cyc	0.062
		+trastuzumab+Vis vs. trastuzumab + Cyc + SMO OE	< 0.001
		+transtuzumab+Vis+SMO OE vs. +trastuzumab+Cyc	< 0.001
		+transtuzumab+Vis+SMO OE vs. +trastuzumab+Cyc+SMO OE	0.069

The descriptive statistics of the Dunn's post hoc and p for each experimental group.

Table S2 Comparison of tumor volumes in nude mice in different experimental groups.

Description	Group	Median	Quartiles (Q1, Q3)
Average tumor volume	NC	0.653	0.635, 0.689
	+Vis	0.342	0.313, 0.371
	+Cyc	0.298	0.277, 0.311

Table S3 Comparison of tumor volumes in nude mice in different experimental groups.

Description	Group	Median	Quartiles (Q1, Q3)
Average tumor volume	NC	0.715	0.688, 0.735
	trastuzumab	0.569	0.524, 0.604
	+trastuzumab+Vis	0.217	0.201, 0.238
	+trastuzumab+ Vis+SMO OE	0.619	0.608, 0.631
	+trastuzumab+ Cyc	0.243	0.224, 0.257
	+trastuzumab+ Cyc+SMO OE	0.533	0.512, 0.545

Table S4 The description of U-Mann-Whitney test.

Figures	Description	Comparison	*p*	*z*
**2G**	Average tumor volume	NC vs. +Vis	< 0.001	3.78
		NC vs. +Cyc	< 0.001	3.78
		+Vis vs. +Cyc	0.016	1.42
**5B**		NC vs. + trastuzumab	< 0.001	3.78
		NC vs. + trastuzumab+ Vis	< 0.001	3.78
		NC vs. + trastuzumab+ Vis+ SMO OE	< 0.001	3.48
		NC vs. + trastuzumab+ Cyc	< 0.001	3.78
		NC vs. + trastuzumab+ Cyc+ SMO OE	< 0.001	3.70
		+trastuzumab vs. + trastuzumab+ Vis	< 0.001	3.78
		+trastuzumab vs. + trastuzumab+ Vis+ SMO OE	0.364	0.91
		+trastuzumab vs. + trastuzumab+ Cyc	< 0.001	3.78
		+trastuzumab vs. + trastuzumab+ Cyc+ SMO OE	0.199	-1.29
		+ trastuzumab+ Vis vs. + trastuzumab+ Vis+ SMO OE	< 0.001	-3.78
		+ trastuzumab+ Vis vs. + trastuzumab+ Cyc	0.010	-2.57
		+ trastuzumab+ Vis vs. + trastuzumab+ Cyc+ SMO OE	< 0.001	-3.78
		+ trastuzumab+ Vis+ SMO OE vs. + trastuzumab+ Cyc	< 0.001	3.78
		+ trastuzumab+ Vis+ SMO OE vs. + trastuzumab+ Cyc+ SMO OE	0.908	-0.11
		+ trastuzumab+ Cyc + SMO OE vs. + trastuzumab+ Cyc+ SMO OE	< 0.001	-3.78

Abbreviations: "p" - significance level; "z" - value of the test statistic.
